# Nonlegacy PCBs: Pigment Manufacturing By-Products Get a Second Look

**DOI:** 10.1289/ehp.121-a86

**Published:** 2013-03-01

**Authors:** Elizabeth Grossman

**Affiliations:** **Elizabeth Grossman**, a Portland, OR–based environ-mental and science writer, has written for *Environmental Health News*, *Yale Environment 360*, *Scientific American*, *The Washington Post*, and other publications. Her books include *Chasing Molecules* and *High Tech Trash*.

Polychlorinated biphenyls (PCBs) were commercially manufactured in the United States from about 1930 until 1979, when their production was banned under the Toxic Substances Control Act (TSCA) because of concerns about their extreme environmental persistence, ability to bioaccumulate, and adverse human health effects. PCBs were used in numerous industrial and consumer applications, most notably as insulation fluids in electrical transformers and generators but also in products including fluorescent lamp ballasts, caulk, and carbonless copy paper. These now-discontinued manufactured chemicals have received a great deal of attention in terms of research and environmental remediation. But other, lesser-known PCBs continue to be generated and released into the environment, not from intentionally created commercial products but as unintentional by-products of manufacturing processes including, according to recent studies, those used to make certain pigments used in dyes, inks, and paints.

PCBs do not occur naturally, and once in the environment they can last for decades. Until recently, PCBs that were being detected in the environment were thought to come entirely from “legacy” sources. Yet developments in analytical technology have given researchers a better understanding of PCB sources, of the patterns of individual PCBs (or congeners) that are being detected environmentally, and the fate of PCBs in the environment—how they move between soil, sediment, water, and air. These advances have also enabled the detection of individual congeners at very low levels and the identification of many new and ongoing sources of PCBs beyond those resulting from historical commercial mixtures.

Unintentionally produced PCBs were known to be present in inks and dyes when the U.S. Environmental Protection Agency (EPA) announced the final rule barring commercial PCB production in 1979. A rule allowing exemptions for PCBs in controlled manufacturing processes and as unintentional contaminants was promulgated under TSCA a few years later. This rule allowed for PCB concentrations of up to 50 ppm in certain products as a result of manufacturing processes.^1^

Recently, manufacturing by-product PCBs have been identified in wastewater, sediments, and air in numerous locations. They have also been positively identified in testing of new products colored with such pigments, so it is clear these PCBs are not occurring as a result of legacy commercial mixtures. What is emerging is an increasingly complex picture of the prevalence of nonlegacy PCBs alongside the persisting environmental presence of legacy PCBs, and a concurrent and likewise complex picture of how PCBs can affect human health at very low levels of exposure.

## PCBs: Products and By-Products

Between 1929 and 1989 an estimated 1.7 million metric tons of PCBs were produced commercially worldwide.^2^ These commercial PCBs were sold in the United States under dozens of different trade names, of which the most commonly known was Aroclor. These products were mixtures that contained different PCB congeners, each with between 1 and 10 chlorine atoms.^3^ (Unintentionally produced PCBs also typically occur as mixtures of congeners.) Each of the total 209 PCB congeners has a distinct structure that influences how it behaves environmentally and how it interacts with living cells and organisms.

Although large amounts of commercially produced PCBs remain in the environment, monitoring conducted at a number of sites around North America in the past five years or so has detected notable air concentrations of PCB congeners that are not part of the historical, now-banned commercial PCB products. These congeners have also been detected in U.S. water bodies including the Delaware River, the New York–New Jersey Harbor, the Houston Shipping Channel, and San Francisco Bay. In the Spokane River in Washington State, such PCBs are currently responsible for violating local water quality standards.^4^

One of these congeners, PCB 11, is emerging as a marker of nonlegacy PCB contamination. In what amounts to environ-mental and scientific detective work, researchers who detected PCB 11 and other congeners in air samples discovered that these PCBs matched those previously detected in water discharged from paint manufacturing facilities. Testing of paint samples has turned up 50 different PCB congeners, including PCB 11, which is known to be produced in the manufacture of diarylide yellow pigments. PCB 11 is neither associated with historical commercial PCB products nor a breakdown or a dechlorination product of those old commercial mixtures.^5^ So if PCB 11 is being found in the environment, it is most likely coming from sources other than legacy PCBs.^6^

PCB 11 has been found in Great Lakes sediment^7^ and in polar region air samples.^8^ Work by a number of research teams points to paints and pigments as likely sources of the PCB 11 being detected environ-mentally.^7^ But PCB 11 is far from the only congener being found in testing of pigments and the products they are used in. Such testing has revealed PCBs ranging from those with just a few chlorine atoms, PCBs 1 through 11, to the most chlorinated congeners, PCBs 206 through 209. These congeners include a number of those previously identified as dioxin-like.^11^ Dioxin-like PCBs are structurally similar to polychlorinated dibenzo-*p*-dioxins and dibenzofurans, chemicals known to be environmentally persistent and to have adverse health effects, among them cancer and impacts on development, reproduction, and immunity. Biologically, these chemicals share an ability to bind to a cellular receptor known as the aryl hydrocarbon receptor, which regulates the expression of genes that influence multiple functions, including enzyme production and hormone regulation.

Recent testing of pigments in Japan by the Japan Dyestuff and Industrial Chemicals Association has found traces of PCBs in 57 out of 98 organic pigments tested.^9^^,^^10^ Some of these pigments were found to contain PCBs at concentrations above 50 ppm. Testing of newspaper and magazine papers, food packaging, and plastic bags colored or printed with inks and pigments associated with PCB by-products has also confirmed the presence of these PCBs, including PCB 11.^5^

The PCBs detected in paint and pigment samples and those detected in finished products colored with such pigments also overlap with those identified in 2012 testing of effluent from the Inland Empire Paper (IEP) Company recycling facility in Spokane, according to IEP environmental manager Doug Krapas. IEP was able to connect the PCBs in its wastewater with inks on the paper it recycles, much of it newspapers, magazines, mailing materials, and packaging. Papermaking processes historically have been a source of chlorine compounds that are known to have adverse health effects on aquatic ecosystems, but over time IEP had changed its processes to eliminate the use of chlorine. It had also installed secondary and tertiary treatment and closed-loop systems to reduce overall water use and water discharges and to reduce contaminants in its wastewater as much as technically possible. When the company discovered that, despite all of these measures, PCBs exceeding local standards were being found it its wastewater, it realized that something in the paper being recycled must be causing the problem.

According to Krapas, as long as recycling facilities continue to process paper that contains ink with allowable levels of by-product PCBs, there will be problems meeting water quality standards. Inkborne PCBs present a particular problem for IEP’s Spokane plant, given the low levels of PCBs allowed by local water quality standards—local Spokane Tribe water quality standards limit PCBs to 3.37 parts per quadrillion (ppq).^12^ This standard is based on fish consumption—that is, the amount of PCBs people would ingest by eating fish from the Spokane River—and is based on the tribe’s reliance on local fish as a staple food.

Keri Hornbuckle, a University of Iowa professor of civil and environmental engineering, says that prior to 2007 most environmental monitoring for PCBs was not designed to sample for all 209 PCB congeners but focused only on those that were produced intentionally, the commercial Aroclor mixtures. This means that environmental monitoring was likely missing detection of PCBs that are unintentional by-products. New monitoring technology used by Hornbuckle and colleagues through funding from the Superfund Research Program of the National Institute of Environmental Health Sciences—initially to monitor air in Chicago but since conducted in many other locations—was what for the first time profiled the environmental presence of unintentionally created PCBs.

Hornbuckle and her colleague Dingfei Hu, a University of Iowa assistant hydroscience and engineering research scientist, hypothesized that because the PCB congeners they were detecting in air samples were the same as those that had been detected in water discharged from paint manufacturing facilities, these PCB congeners might also be present in commercial paints. To investigate, the researchers measured PCBs in paint pigments purchased on the U.S. retail market in 2009.^6^ Their analysis found more than 50 different PCB congeners (including several previously identified as dioxin-like) in the pigments. The identified PCB congeners appeared to vary depending on the types of pigments analyzed and the manufacturing processes involved in their production. Although the PCBs generated as by-products vary by pigment type, the processes that result in these PCBs typically combine chlorine, salts, and hydrocarbons or chlorinated hydrocarbon compounds at high temperatures.

Further analysis indicated that certain PCBs were prevalent in what are called azo, diarylide, and phthalocyanine pigments, which are commonly used to color inks, dyes, paint, paper, textiles, plastics, leather, cosmetics, and foods, among other materials and products. Azo and diarylide pigments are used primarily to make yellows but also some reds and oranges, while phthalocyanine pigments are used primarily to make blues and greens.

“The widespread use of these pigments explains the presence of PCB 11 in commercial goods common throughout modern society, such as newspapers, magazines, and cardboard boxes,” wrote Hu and Horn-buckle. “Although we do not know if inadvertent PCBs have adverse effects on human health, there are many potential routes for human exposure to these PCBs through inhalation, dermal exposure, and ingestion due to their physicochemical characteristics of semi-volatility, hydrophobicity, and persistence.” They also say that to their knowledge, “pigments or dyes are the only significant source of PCB 11,” and therefore detection of PCB 11 in air “must be associated with human activity utilizing pigments or dyes.”^6^

In a subsequent published study, Hu and Hornbuckle noted that the pattern of PCBs detected in the commercial paint pigments tested was unrelated to that of commercial PCB production and included “many congeners that are highly bioaccumulative, dioxin-like and/or probably carcinogens.”^7^ They cited paint production and use as the probable source of these PCBs in North America. Their investigation, which focused on Great Lakes sediment, appeared to confirm the presence in the environment of manufacturing by-product PCBs not associated with historical commercial mixtures. They do note, however, that because their sediment study did not enable them to identify sources of PCB congeners that appear to be common to both legacy commercial Aroclor mixtures and pigment by-products, it leaves important questions unanswered about dioxin-like PCB congeners found in these samples, which have potential multiple sources,

Further establishing the link between pigments and PCBs in the environment is research by Lisa Rodenburg, a Rutgers University associate professor of environmental science, in which she directly tested products printed or colored with pigments associated with by-product PCBs.^5^^,^^13^ Among the printed products Rodenburg tested were colored newspapers, glossy magazine papers, plastic bags, and cereal and other food boxes. The PCBs she identified in these printed paper and plastic samples, primarily those colored yellow, were the same nonlegacy congeners found in environmental samples and in paint pigments by Hu and Hornbuckle. They also matched the PCBs being measured in effluent from IEP’s Spokane recycling facility.

In addition to organic pigments, Rodenburg’s research indicates that certain titanium dioxide manufacturing processes can also produce PCB by-products.^13^ Although Hu and Hornbuckle did not find PCBs in their testing of inorganic pigments, including those containing titanium dioxide, titanium dioxide manufacture has been cited as a source of by-product PCBs by the Michigan Department of Environ-mental Quality,^14^ the New York Academy of Sciences,^15^ and the Australian government,^16^ among others.

Hu and Hornbuckle’s research suggested several possible ways in which by-product PCBs are created during manufacturing. The phthalocyanine blues and greens analyzed so far have tended to contain higher-chlorinated PCB congeners (those with more chlorines) than the diarylide yellows tested. As described by Hu and Hornbuckle, the process commonly used to produce these blues and greens involves phthalic anhydride, urea, or phthalonitrile and a copper or copper salt, which are then processed using an organochlorine solvent such as di- or trichlorobenzene.^6^ As noted above, the combination of chlorinated hydrocarbons and salts processed at certain high temperatures is what leads to the creation of PCBs.

Hornbuckle also explains that the highly chlorinated PCB by-products in blue and green pigments behave differently than the lighter-weight, less chlorinated by-products in the yellow pigments. The lighter, less chlorinated chemicals are more volatile and therefore more mobile in air and can be expected to move out of the pigmented paints and inks more readily than heavier, more chlorinated chemicals would. The heavier blue and green pigment by-products are likely to stay with paints when they chip, thus enabling the by-product PCBs to transfer to soil if these paints are used outdoors—on building exteriors, for example.

According to research by Delaware River Basin Commission geologist Gregory Cavallo along with Rodenburg and colleagues at Rutgers University, about 65% of all organic pigments produced go into printing inks (as opposed to dyes, paints, or other colored products). In 2006 one-quarter of the worldwide production of 250 million metric tons of organic pigments was estimated to be diarylide yellow pigments. Analysis of printing inks indicates that the typical printing ink contains an estimated 40% pigment. Cavallo et al. therefore estimate that worldwide production of PCB 11 through the manufacture of diarylide yellows would have been about 1.5 metric tons in 2006.^5^

## Does Exposure to These PCBs Matter?

The chemical stability of PCBs that made them so useful as industrial products also makes them environmentally persistent and gives them properties that pose potentially serious health hazards. In addition to being slow to biodegrade, PCBs are lipophilic (fat soluble) so they can bioaccumulate and move up the food web. Certain PCBs have been identified as carcinogens.^17^

PCBs have also been identified as endocrine disruptors and shown to have adverse effects on the endocrine system, particularly on thyroid hormone function. They are also associated with skin and eye problems, liver toxicity, and adverse effects on the immune, nervous, and reproductive systems as well as on blood pressure and blood cholesterol levels. Prenatal and childhood PCB exposure has been associated with behavioral and cognitive problems. Among the PCB health effects now under investigation are their impacts on brain functions that control behavior, language, learning, and memory.^17^

Some nondioxin-like PCB congeners have been recognized as having what R. Thomas Zoeller, a University of Massachusetts professor of biology, calls “potent” effects on body systems regulated by the thyroid, at very low levels of exposure—those measured in micrograms per kilogram, or parts per billion. Thyroid hormones are involved with regulating a number of vital body systems, including metabolism and development, and they are essential for healthy cardiovascular and nervous system function. If PCBs can interfere with thyroid hormones, “no one can conclude that PCBs are safe,” says Zoeller.

Larry Robertson, a University of Iowa professor of environmental and occupational health, explains that since PCBs typically occur as mixtures of congeners, it is important to note that an individual congener’s health effects should not be excluded when considering PCB toxicity, even if that congener is not dominant in a particular sample or mixture. “Context is everything,” he says of health effects.

That PCBs have endocrine-disrupting effects is important to understanding exposure levels that may affect human health, as it is recognized that such chemicals can be biologically active at extremely low levels.^18^ It is also recognized that timing of exposure to endocrine disruptors is important, as exposure at one life stage can prompt effects different from those of exposure at another time. Early life, including prenatal development, is a time at which many body systems are particularly vulnerable to effects of endocrine disruptors.^19^

Zoeller says that thyroid hormone levels at age 2 have been correlated to adult IQ levels, and early-life PCB exposures have been associated with depressed IQ scores and impaired cognitive ability in children.^20^ He also says that studies have shown metabolites of PCB 105 and PCB 118 to be specifically related to cognitive function in animals.^21^^,^^22^ Furthermore, research by Isaac Pessah, chair of molecular biosciences at the University of California, Davis, School of Veterinary Medicine, has shown that neural network formation was adversely affected in rats exposed prenatally to PCB 95.^23^^,^^24^

The task of trying to assess all 209 PCB congeners and their metabolites is enormous, Pessah says, so he and his colleagues decided to look for certain cellular targets that appear to be extremely sensitive to PCBs. “The one we found that was most active was that activated by PCB 95,” he says. This congener has been found in sediment and in fish and human tissue, including children’s brain tissue.

Pessah says that the cellular receptor with which PCB 95 interacts—the ryanodine receptor—is similar to the aryl hydrocarbon receptor to which dioxin-like PCBs are known to bind. If a PCB or dioxin binds to the ryanodine receptor, it may cause the production of certain proteins that can interfere with normal cellular growth and differentiation, he explains. The interaction of PCB 95 with the ryanodine receptor, which is known to play a critical role in regulating the movement of calcium around the body,^23^^,^^24^^,^^25^ could be very important, as calcium is key to healthy neuron function.

“The brain is very organized,” Zoeller says, a characteristic integral to how it works. It appears from Pessah’s animal experiments that prenatal exposure to PCB 95 can interfere with this organization and disrupt the brain’s auditory perception, a function that is vital to speech and language abilities. Curiously, there were no overt signs that there was anything wrong with the exposed animals, Pessah says—they didn’t fail to thrive, and they could still hear—but their ability to interpret sound was impaired. These effects, he says, have potentially profound implications for PCB toxicology, for it may mean that a level of exposure that does not otherwise appear “toxic” could nevertheless change the course of neural development.

**Figure:**
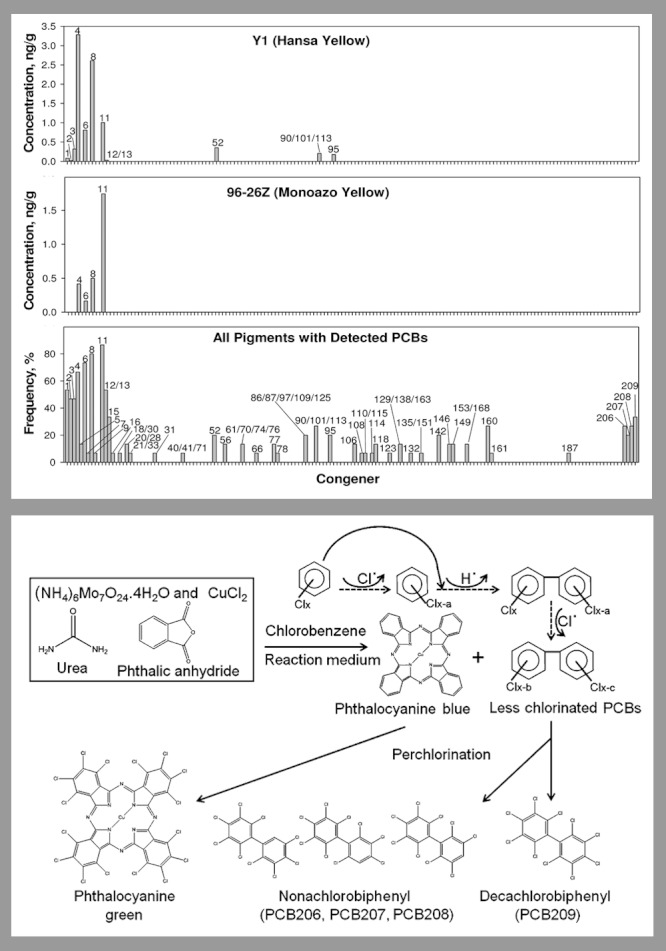
In a study of 33 commercially available paint pigments,6 Hu and Hornbuckle depicted PCB profiles for two specific yellow pigments as well as the frequency of congener detection across all 15 samples in which PCBs were detected (above). The authors also proposed potential mechanisms for PCB formation during the manufacture of phthalocyanine pigments (below). As shown, phthalocyanine green is derived from phthalocyanine blue via chlorination.

Another recent but very different type of study, an epidemiological study examining the relationship between environmental chemical exposure and fertility in couples, has found an association between exposure to certain PCB congeners—among them PCBs 118, 138, 167, and 209—and the reduced likelihood of becoming pregnant within a certain time.^26^ Study author Germaine Buck Louis, director and senior investigator at the Eunice Kennedy Shriver National Institute of Child Health and Human Development’s Division of Epidemiology and Prevention Research, cautions that “we cannot say if anything is causal.” But she says, these are “important signals that are being picked up,” particularly as this study indicates an association between environmental chemical exposure in both men and women and a delay in the time to successful conception and pregnancy.

In addition to the health effects of PCBs themselves, there is also evidence that some PCBs can, once in the body, break down into other compounds that can play a role in health effects. Among these compounds are hydroxylated PCB metabolites (OH-PCBs)—some of which have structures that resemble a thyroid hormone—and PCB sulfates, which can also be endocrine disruptors. Robertson says there could be as many as 15 or 20 different metabolites of a single parent PCB compound, depending on how that compound is metabolized. What appears to be emerging from the growing body of research on PCB health effects is an increasingly complex picture of the ways in which various PCB congeners and their metabolites can affect human health—potential effects that extend well beyond those of the dioxin-like and carcinogenic PCBs that have received the most research and remediation attention to date.

## Regulatory Considerations

By-product PCBs have become a matter of concern to state governments working to meet water quality and fish consumption standards for PCBs, some of which are far more stringent than those set by the EPA. For example, as noted above, the Spokane Tribe’s water quality standards for PCBs based on fish consumption in Washington are more than 95% lower than those set by the EPA. The federal government has set this limit at 170 ppq, but the Spokane Tribe set a standard of 3.37 ppq, reflecting that local population’s high fish consumption.^12^ Although state and local governments have been working to address legacy PCB issues, the nonlegacy PCB issue has emerged as paper recycling has increased nationwide and begun to be recognized as an ongoing PCB source. At its 2012 summer meeting, the Environmental Council of the States (ECOS), an association of state environmental agency leaders, passed a resolution calling on the EPA to work with industry and states to develop cleaner pigment and ink manufacturing processes and products.^27^

As illustrated by the case of the IEP recycling facility—which has no chlorinated waste sources apart from the paper it recycles—the amounts of PCBs allowable in printing ink pigments may make it impossible for facilities to meet local water quality standards for PCBs, even with state-of-the-art water treatment.^4^^,^^12^^,^^28^^,^^29^ As long as IEP continues to take in paper printed with PCB-containing inks, these compounds will remain in its waste stream. ECOS would like to see these contaminants controlled upstream at the product and process source rather than making this the full responsibility of facilities at the downstream end of the product life cycle.

This is an issue of which EPA is well aware. In April 2010 the agency published an advanced notice that it intends to reassess its PCB regulations.^30^ Among its proposals is a revision of the current definition of an “excluded manufacturing process” that permits certain by-product PCBs up to 50 ppm. The proposed revised rule would eliminate the limit on the allowed annual average concentration of PCBs and reduce the maximum PCB concentration allowed in manufactured or imported products to less than 1 ppm. This overall rulemaking process began in 2009 and is currently not projected to be completed until 2014. When asked in December 2012 for details on its status an EPA spokesperson would not provide any details, but said the agency is “still considering comments and working on the rule.”

In a 2010 letter to the EPA submitted as part of the rulemaking docket, the Color Pigments Manufacturing Association (CPMA), a trade association representing companies in the United States, Canada, and Mexico, stated that the manufacturing processes involved in making diarylide, phthalocyanine, and certain monazo pigments would be negatively impacted if by-product PCBs were disallowed from these products. CPMA said it is not technically feasible to alter manufacturing processes to eliminate by-product PCBs or to reduce them to the proposed 1-ppm level, nor is testing to such a level feasible.^31^

The industry association also said that taking these pigments off the market would jeopardize most color printing, the vast majority of yellow, blue, and green paint as well as many plastic formulations, and a large number of red pigments used primarily in paints, plastics, and specialized inks.^31^ Furthermore, the association claimed, compliance with the proposed 1-ppm level would put the U.S. industry at a competitive disadvantage internationally—a comment the EPA has noted in responses to inquiries about the rulemaking.^32^^,^^33^

Neither CPMA nor any companies that use pigments in inks, dyes, or paints contacted for this story would discuss manufacturing processes directly or provide details of why changing current processes is so challenging. However, John Warner, president and chief technology officer of the Warner Babcock Institute for Green Chemistry in Wilmington, Massachusetts, who holds more than 30 chemical patents, explains that to be commercially successful, a pigment has to be persistent. It has to be stable in light and, depending on the application, also stable in water. Also depending on the application, the pigment may have to be compatible with adhesives or have adhesive properties itself. This, says Warner, explains why come commercial pigments are manufactured with processes that would also produce environmentally persistent by-products.

In its comments to the EPA, CPMA also said that pigments that contain PCB by-products are not toxic or bioaccumulative.^31^ In a January 2011 position paper on this issue, the Ecological and Toxicological Association of Dyes and Organic Pigment Manufacturers (ETAD), a trade association based in Switzerland, wrote that trace levels of by-product PCBs in pigments do not pose a human health hazard. ETAD said pigments are incorporated into products in such a way that environmental or human exposure to these PCBs is unlikely to occur and that release of any PCBs is “improbable—until both polymeric matrix and the pigments degrade.” ETAD further stated that PCBs in wastewater would likely be captured by filtration, although it does not have any data to support that speculation. It also said in the same document that there is no information to link PCB 11 with pigments.^34^

Asked to comment on these assertions, Robertson says that PCBs “do not degrade even if the matrices in which they may be applied do.” He also noted that PCBs can be released from dyes and pigments and become a major concern in city wastewater, as the Rodenburg research shows. To say that PCBs “are trapped and do not leave the matrix, that is clearly false,” he says.

As part of its comments to the EPA on the proposed revision of allowable by-product PCB levels, CPMA submitted a 1987 study conducted by researchers affiliated with ETAD and chemical manufacturer Ciba-Geigy, which had a long history of dye manufacturing. The study, which looked at the relative solubility in water and octanol of various organic dyes, concluded that since organic pigments are not readily soluble in these media and are of large molecular size, they are unlikely to be taken up by fish and therefore do not have to be assessed for bioaccumulation in fish.^35^ But Robertson points out that the dyes tested in the study have many functional chemical groups that allow for degradation by microbes and other organisms. This, he says, may mean that once in an aquatic environment, breakdown products might predominate. He also notes that PCBs do not readily biodegrade in water, so if they are present in pigments or dyes released to water, they might persist after the rest of the compound breaks down.

Also important to understanding by-product PCBs’ presence in the environment is the fact that different environmental monitoring and testing methods will detect different PCB congeners. Some methods are more sensitive than others and are designed to more precisely detect and identify individual PCB congeners.^36^ According to IEP environmental manager Krapas, the test methods approved by the EPA for regulatory purposes are not the most sophisticated available. What this means is that if testing is done using only the PCB monitoring tests currently used to meet EPA regulatory requirements, some congeners—including those in products such as inks and pigments—could remain undetected.

## The Picture Thus Far

What we know thus far is that a range of PCB congeners are created as by-products in the current manufacturing processes for certain pigments. Some of these PCB congeners are being found in the environment, in air, water, and sediment samples. These PCBs have been found in testing of pigmented products, printed papers, plastics, and paints. They have also been found in wastewater resulting from recycling paper printed with such pigments.

It is also known that the level at which by-product PCBs are currently allowable under U.S. law exceeds standards set by other entities for PCBs in certain water bodies and fish. In some locations, these standards cannot be achieved with currently available water treatment technology as long as what’s going into the waste stream includes new sources of PCBs at levels measured in products such as paints, inks, and pigments.

Recent research has yielded increasing evidence that certain PCB congeners not historically studied for adverse health effects appear to have endocrine-disrupting effects and/or the ability to interfere with the function of cellular receptors that play a vital role in regulating neural development and function. There is also evidence suggesting that PCBs should be examined for their potential to affect fertility. Some of the PCB congeners associated with these end points are common to the list of congeners identified in pigmented products and detected environmentally.

While efforts have been made to remove heavy metals and volatile organic compounds from various pigmented products—paints, for example—environmental and materials sustainability experts say little attention has been focused thus far on the issue of by-product PCBs. Whether it is feasible to alter the pigment production processes that result in by-product PCBs cannot be fully explored without the cooperation of manufacturers. Multiple contacts to the industry yielded little information beyond the fact that pigmented and dyed products have complex supply chains that vary by specific product. Chemical management and environmental consultants described pigments as a difficult product category to assess due to the highly proprietary nature of product formulations.

The EPA’s Design for the Environment Program, which is conducting alternative assessments on various chemical products, is not currently involved in any research on pigments. That the EPA initiate such research is one of the recommendations put forth in the ECOS resolution.^27^ As rulemaking continues for the EPA’s reassessment of current PCB regulations, an EPA spokeswoman provided no information on the status of the agency’s ongoing assessment of non-dioxinlike health effects of PCBs.

Although questions remain about the potential human health effects of the PCB congeners associated with manufacturing by-products, the presence of these compounds at sites across North America is worrisome, given what is known about PCBs overall and given that, as ECOS notes, PCB-contaminated fish continue to be a primary source of human exposure to these chemicals, with 1,084 fish advisories for PCBs issued by 40 states in 2010.^37^ Among the remaining questions is how exposure to these PCBs may act in combination with exposure to legacy PCBs and other chemicals with similar potential for endocrine-disrupting activity.

At the same time, a growing body of scientific research is emerging to suggest that low-level exposure to some of these PCBs may have potentially profound effects on biological mechanisms vital to healthy development. What is being learned about the potential health effects of the less-well studied PCBs, says Zoeller, could yield important information about the role these chemicals might play in increasing prevalent chronic diseases.
